# Breeding of Vegetable Cowpea for Nutrition and Climate Resilience in Sub-Saharan Africa: Progress, Opportunities, and Challenges

**DOI:** 10.3390/plants11121583

**Published:** 2022-06-15

**Authors:** Tesfaye Walle Mekonnen, Abe Shegro Gerrano, Ntombokulunga Wedy Mbuma, Maryke Tine Labuschagne

**Affiliations:** 1 Department of Plant Sciences, University of the Free State, Bloemfontein 9301, South Africa; mbumanw@ufs.ac.za (N.W.M.); labuscm@ufs.ac.za (M.T.L.); 2 Agricultural Research Council-Vegetable, Industrial and Medicinal Plants, Pretoria 0001, South Africa; agerrano@arc.agric.za; 3 Food Security and Safety Focus Area, Faculty of Natural and Agricultural Sciences, North-West University, Mmabatho 2735, South Africa

**Keywords:** climate change, cowpea, food, gene pyramiding, nutrition security, speed breeding

## Abstract

Currently, the world population is increasing, and humanity is facing food and nutritional scarcity. Climate change and variability are a major threat to global food and nutritional security, reducing crop productivity in the tropical and subtropical regions of the globe. Cowpea has the potential to make a significant contribution to global food and nutritional security. In addition, it can be part of a sustainable food system, being a genetic resource for future crop improvement, contributing to resilience and improving agricultural sustainability under climate change conditions. In malnutrition prone regions of sub-Saharan Africa (SSA) countries, cowpea has become a strategic dryland legume crop for addressing food insecurity and malnutrition. Therefore, this review aims to assess the contribution of cowpea to SSA countries as a climate-resilient crop and the existing production challenges and perspectives. Cowpea leaves and immature pods are rich in diverse nutrients, with high levels of protein, vitamins, macro and micronutrients, minerals, fiber, and carbohydrates compared to its grain. In addition, cowpea is truly a multifunctional crop for maintaining good health and for reducing non-communicable human diseases. However, as a leafy vegetable, cowpea has not been researched and promoted sufficiently because it has not been promoted as a food security crop due to its low yield potential, susceptibility to biotic and abiotic stresses, quality assurance issues, policy regulation, and cultural beliefs (it is considered a livestock feed). The development of superior cowpea as a leafy vegetable can be approached in different ways, such as conventional breeding and gene stacking, speed breeding, mutation breeding, space breeding, demand-led breeding, a pan-omics approach, and local government policies. The successful breeding of cowpea genotypes that are high-yielding with a good nutritional value as well as having resistance to biotics and tolerant to abiotic stress could also be used to address food security and malnutrition-related challenges in sub-Saharan Africa.

## 1. Introduction

Cowpea [*Vigna unguiculata* (L.) Walp.] is a nutritious, underutilized vegetable legume crop that has the potential to alleviate protein-calorie malnutrition [1]. The crop can be grown under harsh conditions such as drought and sandy soils. Thus, its ability to tolerate climate change makes it an important legume crop for food and nutritional security in tropical and sub-tropical regions of the world, including in sub-Saharan Africa (SSA) [1,2,3,4]. Cowpea is known in its dry grain form as black-eyed pea, southern bean, China pea and marble pea, and in its green pod form as a yard-long bean, an asparagus bean, a body bean and a snake bean [5,6]. The hulls of cowpea are fed to livestock as a nutritious supplement to cereal fodder [7]. Cowpea contributes to the sustainable improvement of the environment [8] due to its biological nitrogen fixation ability and its positive effects on the soil [9], as it increases microbial diversity in the soil and plays a major role in resilience to current climate changes in the region and beyond. Due to the high protein content in its leaves, immature pods and grain, cowpea is a potential crop for reducing the deficiency in plant-based protein production in SSA [10]. Despite its role in the food system value chain, there is limited information documented for future sustainable production and improvement of this crop for nutritional diversity and on utilization and key production constraints that affect the performance of the crop in SSA.

## 2. Use of Cowpea as a Leafy Vegetable and Grain Crop

It has been reported that about 150 million children in the world under five suffered from stunting in 2020. Forty-five million of these children were wasted. Approximately 39 million children globally under five years of age are overweight [11]. Childhood obesity is also currently challenging and is affecting the economy and development of the world population [12]. It has been reported that one in four children in South Africa suffers from severe acute malnutrition (SAM), which remains a fundamental severe cause of child morbidity [13]. The South African Child Gauge report indicated that 44% of children under the age of five suffer from vitamin A deficiency, and 27% are stunted [13]. Meanwhile, 33.3% of women aged 15–49 years are anemic, while 31.3% of males (≥15 years) and 67.6% of females (≥15 years) are either overweight or obese [14]. Regarding food security, about 56% of the population in South Africa live in poverty and almost 28% live in extreme poverty [15]. Several nutrition-sensitive approaches are being introduced in response to malnutrition and the food insecurity burden [16].

Cowpea is an underutilized plant species that can contribute to food, nutrition, and health security in SSA. Cowpea is consumed in many forms; its young leaves, growing points, green pods, and green seeds are all used as vegetables in SSA [10]. Dry seeds are used in various food preparations that have been recommended for the possible alleviation of food and nutrition insecurity [10]. Cowpea leaves can be dried and used as a meat substitute in the rural areas of Africa. Cowpea flour provides extra protein and nutrition that enhances the growth and development of healthy children.

The fresh and young leaves of cowpea, which is ranked among the top four vegetables in 24 countries in Africa and seven in Asia, are suited for production in high rainfall agro-ecologies [17]. Hence, the use of cowpea as both a vegetable and grain crop can contribute to reduced malnutrition in the region.

**Figure 1:**
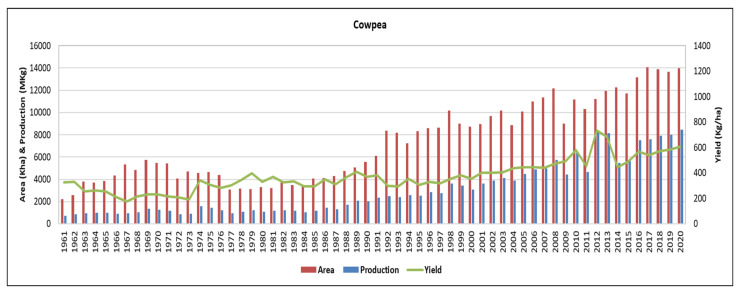
Trends in area, yields and production of cowpea in SSA. The source adapted from [19].

Globally, cowpea production has been reported to have increased by 88% and yields have increased by 35% [18]. The total cowpea area harvested has also risen by 38% in the same production period [19]. This increase in area, production, and yield has been made possible by a similar trend in SSA, which dominates the world scene [20]. In the last three decades, cowpea’s area coverage and production potential has increased significantly (Figure 1) [20]. Sub-Saharan Africa dominates cowpea production, with a 96% (4.9 million tons) area share [19,21]. Total area, yield, and production in SSA have grown at the rate of about 4.3%, 1.5%, and 5.8%, respectively [19,20]. Generally, the area under cowpea has increased, with many developing countries contributing to the total production from the last decade onwards [19]. Over 95% of the global production is in Africa, especially in SSA, with Nigeria being the world’s largest producer and consumer, followed by the Niger Republic and Burkina Faso [22].

In sub-Saharan Africa, the major legume crops in the region are groundnuts, chickpea, pigeonpea, common bean, cowpea, and soybean. In SSA, the area coverage was about 36 million hectares (about 16.3% of global area), with production of about 27 million metric tons (around 6% of global production) at an average productivity of 0.89 metric tons (MT) per hectare (beans = 0.94, chickpea = 0.98, cowpea = 0.48, groundnut = 0.96, pigeon pea = 0.86, soybean = 1.01) [20,21]. The SSA region accounted for more than 99% of the cowpea area and 96% of production, with an average yield of about 0.52 MT per ha in 2020 [19,21]. Cowpea production in SSA is projected to grow from about 9.8 million MT in 2020 to nearly 12.3 million MT by 2030 [19,21].

## 3. Constraints to Cowpea Production

Climate change and variability trends affect crop yields both directly and indirectly [23]. Direct impacts include effects caused by a modification of physical characteristics such as low or high atmospheric temperature, soil fertility level, and water deficiency or erratic rainfall distribution in specific crop production systems [24]. Indirect effects are those that affect production through changes in other species such as pollinators, insect pests, diseases, and weeds. These indirect effects can play a significant role in the production and productivity of cowpea [24]. These limiting factors can broadly be termed abiotic and biotic stresses, resulting in climatic variations and ultimately reducing cowpea yield potential and its productivity [25,26,27].

### 3.1. Biotic Stresses

#### 3.1.1. Bacterial Diseases

Bacterial disease is the most economically important biotic stress in cowpea production in SSA countries. Among them, cowpea bacterial blight (CoBB) and bacterial pustules are some of the most severe bacterial infections of cowpea, causing severe damage [26,27]. CoBB symptoms start with small water-soaked spots on leaves, which enlarge to irregular brown necrotic lesions surrounded by yellow haloes, leading to premature leaf drop [26]. The pathogen also invades the cowpea stem, causing canker symptoms in susceptible plants [27]. It generally infects all growth stages of the cowpea plant: seedling, vegetative, flowering, and podding stages, and all plant parts, including leaves, pods, and seeds [28]. Frequent evaluation of cowpea genetic materials and resistant line development is the best option for achieving desirable resistance gene sources for use in bacterial resistance through breeding programs.

#### 3.1.2. Viral Diseases

Globally, cowpea is infected by more than 140 viruses, but only nine were reported as economically important [29]. Cowpea aphid-borne mosaic virus (CABMV), cowpea golden mosaic virus (CPGMV), Southern bean mosaic virus (SBMV), Sunhemp mosaic virus (SHMV), blackeye mosaic virus (BICMV), cucumber mosaic virus (CMV), cowpea mottle virus (CMV), cowpea yellow mosaic virus (CPMV), and cowpea mild mottle virus (CPMMV) are the most common seed-borne viral diseases [30]. Viral diseases can cause a yield loss of 10% to 100%, depending on the time and the severity of infection in cowpea [31]. The aphids suck the cowpea sap, which might affect the physiological processes and mineral element transportation in the plant system and consequently affect its concentration [10]. It is also a vector in the transmission of viruses [32]. For tackle the existing viral disease problem, intensive evaluation of cowpea genotypes with multiple viral infections under greenhouse and hotspot production areas are the best option to develop resistant varieties. In addition, using biotechnology that will result in the production and deregulation of virus-resistant cowpeas through coat-protein gene transfers should be intensified [29].

#### 3.1.3. Root-Knot Nematode

Root-knot nematode, *Meloidogyne incognita*, is a severe pest and a major constraint on cowpea production in most growing areas of the world, causing 80 to 100% yield losses [32,33,34,35,36,37,38,39,40,41,42,43,44,45,46,47,48]. *M. incognita* and *M. javanica* are the major species found on cowpea in most growing regions [33]. Damage symptoms of root-knot nematodes include patches of stunted and yellowed plants. Severe damage can lead to reduced numbers of leaves and buds [5].

#### 3.1.4. Parasitic Weeds

Parasitic weeds, *Striga gesnerioides* (Willd) Vatke ex Engl and *Alectra vogelii* (Bent), are serious threats to cowpea production in Africa [31,32]. Weeds reduce cowpea yield and quality by competing for light, space, water, soil nutrients, and carbon dioxide [33,34]. In total, yield losses caused by parasitic weed infestation alone in cowpea ranges from 73 to 100% [34,35,47]. These weeds may also reduce productivity by releasing allelopathic compounds into the environment [40] and by providing a conducive environment and serving as a vector for insect pests and viruses [41].

Completely removing these parasitic weeds in cowpea production is too difficult because the seeds can remain viable in the soil for up to 20 years [31,39]. Therefore, the best and most sustainable option to address this critical problem is to breed cowpea varieties which are resistant to these weeds, using multiple smart and agro-biotechnology techniques that could be deployed sustainably.

#### 3.1.5. Insect Pests

Cowpea is attacked and damaged by insect pests in all stages of growth [43]. Insects are the most challenging threat to cowpea production and productivity because they occur at pre-flowering, post-flowering, and storage [44]. Seed com, maggot, cutworm, aphids, and leafhopper occur at the pre-flowering (seedling) stage. Aphids, leaf miners, and thrips are also active insect pests at the flowering and post-flowering stage, and aphids, bean fly, bean pod borer, leaf miner, and thrips are the common insect pests at the reproductive stage (grain filling period) of cowpea [45]. Among the field pests of cowpea, aphid [*Aphis craccivora* (Koch)] is an important vegetative stage pest of cowpea in Africa but also occurs at other growth stages. Both nymphs and adults suck plant sap and cause serious damage from the seedling to the pod bearing stage [46]. Aphids cause damage through secretion of honeydew, which promotes the growth of sooty molds and other fungi on leaves, curling of leaves and delayed flowering, shriveling of pods and, as a result, reduced photosynthetic processes and rates, finally resulting in overall yield reduction [47]. It affects the crop by directly sucking its sap. Their feeding on cowpea causes cupping of the leaves, crinkling, defoliation, and stunted growth [48]. Another serious effect of aphids is the ability to transmit the aphid-borne mosaic virus. Affected plants show a green vein banding of the leaves [49]. Molecular and phenotypic screening of cowpea [35] identified cowpea genotypes with good resistance to aphids, which can be used as a source of resistance genes in breeding new aphid-resistant cultivars.

### 3.2. Abiotic Stress

#### 3.2.1. Drought

Cowpea is known to be drought-tolerant compared to other crop plants [50,51]. Among the abiotic factors, drought has been identified as a significant limitation, restricting cowpea production and productivity [50]. In the arid and semi-arid tropics, the productivity of cowpea could be hampered by erratic rainfall at the beginning and towards the end of the rainy season [52]. Drought leads to adverse influences on cowpea growth, development, and reproduction ability [53], limiting the crop’s yield and productivity [54]. This crop plant is a robust legume; nevertheless, drought always affects yield, especially during the reproductive and seed-filling period [55]. This causes a substantial reduction in grain yield and biomass production [51]. The influence of drought varies and depends on the intensity, developmental stage, and duration of stress and the adaptive strategy that the plant possesses to tolerate this stress [51,56]. Cowpea suffers from drought stress due to erratic rainfall due to climate change, resulting in a yield loss of up to 35–69% [57]. To provide a better solution to drought stress in cowpea production in SSA, a comprehensive evaluation and characterization of cowpea genotypes for developing drought-tolerant varieties using physiological, biochemical, and molecular approaches could be exploited. Understanding drought tolerance gene expression will further advance tolerance breeding.

#### 3.2.2. Salinity

Salt stress is one of the most significant abiotic constraints to cowpea crop productivity and severely hampers crop production, especially in arid and semi-arid areas. Cowpea is unfavorably affected by salinity stress at seed germination and seedling stages, and growth and vigor are reduced, which is exacerbated by climate change effects [58]. Salinity stress ultimately reduces the yield (leaves, immature pods, and grain weight and quality) of cowpea, while other vegetative growth traits are also adversely affected [59,60]. In addition, salinity reduces the ability of cowpea crops to take up water and soil-plant nutrients, leading to growth reduction and metabolic changes similar to those caused by low soil moisture stress [61]. Furthermore, it reduces lipid peroxidation and leads to destructive oxidation, which in turn causes damage to the key plant biomolecules [62]. Salt stress is a complex trait, and it is associated with other agronomic and biochemical traits of cowpea. Therefore, for developing salt stress-tolerant cowpea genotypes, integrative (biochemical, molecular, and conventional) breeding approaches are the best solution for tackling the existing problems in SSA.

#### 3.2.3. Heat Stress

Heat stress is a crucial abiotic stress that significantly affects the growth and yield of cowpea [14]. The effects of heat stress on yield and yield-contributing traits include flower and leaf drop, poor pollen fertility and germination, low pod setting, low plant biomass, low harvest index, poor pod filling, and low seed weight and yield [63,64]. Furthermore, physiological and biochemical traits of cowpea negatively affect the photosynthetic apparatus, such as impaired photo-assimilation, inhibited N2 fixation, increased leaf senescence, decreased canopy temperature, and leaf relative water content [64,65,66]. When the night temperature reaches about 16 °C, cowpea flowers abort due to poor pollen development, which causes a 4 to 14% loss in leaves, immature pods and grain yield and quality [18]. In general, using conventional, physiological, phenomic, functional genomic, proteomic, and metabolomic breeding techniques [66,67,68,69,70] and viable approaches to developing heat stress tolerance and to sustain cowpea yield under increasing high-temperature stress, the cowpea germplasm needs to be screened to identify ‘stress adaptive’ traits across various gene pools.

#### 3.2.4. Low Soil Fertility

Phosphorus is essential for cowpea production in many tropical African soils with the inherent low soil availability of phosphorus [71]. Cowpea does not require too much nitrogen fertilizer because it fixes its nitrogen from the air using the nodules in its roots [72]. However, phosphorus is critical to cowpea yield because it stimulates growth, initiates nodule formation, and influences the efficiency of rhizobium-legume symbiosis [73]. Therefore, cowpea requires more phosphorus than nitrogen in the form of single super phosphate [74]. In addition, it is required in large quantities in young cells such as shoot and root tips where metabolism is high and cell division is rapid [75]. It also aids in flower initiation and seed and fruit development [76].

## 4. Breeding Opportunities and Nutritional Profiles of Cowpea Leaves

Cowpea, in Africa and parts of Asia and the American countries, is called ‘the poor man’s meat’ as it is a significant and cheap source of protein, minerals, and vitamins [77] for rural poor people who have limited access to protein from animal sources such as meat and fish [48,49]. It is a nutritious food source, as it is rich in protein and minerals, digestible and non-digestible carbohydrates, and potassium and has a very low lipid and sodium content [77,78].

Fresh cowpea leaves are used as a vegetable, and the haulms, pods, stems, and leaves are used as livestock fodder, providing dietary nutrients for animals and humans [49,77,79,80]. Moreover, all of these components are high in protein, low in fat, and are a vegetable source for human consumption [11,80].

### 4.1. Protein Quality and Quantity

Cowpea is a dry land legume crop consumed as a high-quality plant-based protein source in many parts of the world, particularly in the tropics [6]. In addition, this crop has been promoted as a high-quality protein part of the daily diet of economically depressed people in developing countries to reduce the high prevalence of protein and energy malnutrition [81,82,83,84,85]. Cowpea grain has relatively low-fat and high total protein content [6]. On average, the protein content of cowpea leaves is between 27 to 43% (Table 1), while that of dry grain is 21 to 33% [80], with a high amount of essential amino acids like lysine and tryptophan [86]. Nevertheless, cowpea protein compared to animal protein is deficient in methionine and cysteine [87].

### 4.2. Minerals

Cowpea grains, leaves and immature pods are a source of essential minerals (Ca, Cu, Fe, K, Kg, Mg, Mn, Na, P and Zn, Al) that are required for human health, growth, and development [84,92]. Moreover, Al, B, and Se are present in cowpea leaves and grain [98]. According to previous studies, the amount and availability of minerals in cowpea leaves and immature pods are much higher than in the grain (Table 2). The availability of some minerals like P, K, and Mn in cowpea grain varies widely due to environmental conditions [78]. Macro and micronutrients are essential for the physiological functions of the human body [94].

### 4.3. Vitamins

Cowpea is a source of different vitamins (Table 3). It is rich in vitamin A and C [99,105,106] and has an appreciable amount of B complex vitamins (thiamine, riboflavin, pantothenic acid, pyridoxine, and folic/folate acid) [84]. The vitamin E composition seems to differ significantly from that of most other legume crops, where γ-tocopherol dominates [86]. Vitamin C composition in cowpea leaves is 4- to 38-fold that of the grain [100].

### 4.4. Fatty Acids/Lipids

Compared to chickpea, lentil, green gram, and lupin, cowpea has a low lipid content [91]. The lipid content of cowpea grain and leaves ranges from 0.5% to 3.9% and 1.3 to 4.3%, respectively (Table 4). The lipid profile of cowpea indicates a predominance of triglycerides (41.2% of the total fat), followed by phospholipids (25.1% of total fat), monoglycerides (10.6% of total fat), free fatty acids (7.9% of total fat), diglycerides (7.8% of total fat), sterols (5.5% of total fat), and hydrocarbons plus sterol esters (2.6% of total fat) [86,102]. Palmitic and linoleic acids predominate, followed by oleic acid, stearic acid, and linolenic acid [49]. The main component in the sterols is stigmasterol (42.1 to 43.3%), followed by β-sitosterol (27.6 to 39.5%). In the tocopherol fraction of cowpea seed oil, γ-tocopherol ranged from 44% to 67%, followed by δ- tocopherol (30.3 to 52.8%) [102]. The oil content in cowpea grain is relatively low. Still, it has an extremely high content of biologically active compounds (tocopherols in the oils range from 3838 to 11475 mg/kg, phospholipids 12.2% to 27.4%) [102].

### 4.5. Carbohydrates

Most of cowpea grains, leaves, and immature pods consist of carbohydrates (Table 4). Cowpea leaves and grain are an excellent source of carbohydrates, ranging between 30.39 to 31.11% [62,105] and 50 to 60%, respectively, which makes cowpea a potentially important nutritional component in the human diet [104]. The carbohydrate fractions in cowpea are sucrose (11 to 19 g/kg), glucose (4 to 5 g/kg), fructose (1 to 2 g/kg), galactose (≤15 g/kg), and maltose (≤11 g/kg). Anti-nutrient components of carbohydrates of cowpea are stachyose (17 to 60 g/kg), verbascose (6 to 13 g/kg), and raffinose (5 to 10 g/kg) [86].

### 4.6. Pharmacological Benefits of Cowpea

Globally, meat-based food systems have some drawbacks compared to a vegetable-based food system because it requires more energy for digestion [106]. Protein from cowpea has good nutritional properties and health benefits [106,107]. Cowpea is also a source of many health-promoting components, such as soluble and insoluble dietary fiber, phenolic compounds, minerals, and other functional compounds, including B complex vitamins and tocopherols, anthocyanins, and carotenoids [105,106,107].

Consumption of cowpea exerts protective effects against several chronic diseases [110,111,112]. Among the health benefits of cowpea are low glycemic index carbohydrates [90], positive effects on gastrointestinal disorders [112], cardiovascular diseases, hypercholesterolemia, and obesity [113], as well as anti-diabetic [114], anti-cancer [115], anti-inflammatory [116], anti-hypertensive and hypocholesterolemic [117] properties, and positive effects on insomnia, osteomalacia, osteoporosis, anencephaly, rickets, and cardiac health and metabolic wellbeing [17]. Furthermore, consumption of cowpea protein has been linked to reducing plasma low-density lipoprotein [118].

## 5. Cowpea as a Climate-Resilient Crop

Climate change is arguably the most severe global challenge facing the planet in the 21st Century, as temperatures continue rising, triggering a host of extreme weather events such as heatwaves, droughts, and flooding [119]. Climate change affects the growth of crops through multiple mechanisms, including changing phenology, heat stress, soil fertility, and water stress (frequent and prolonged drought) [120]. Increasing climate variability is, arguably, one of the greatest challenges to food security, particularly through its effects on the livelihoods of low-income people in marginalized communities, which have less capacity for coping, and who depend on highly climate-sensitive crop production activities typical of developing countries [121]. Climate change, along with current agricultural practices, is going to pose a significant challenge for future food and nutrition security, particularly in developing countries [122].

Food security is the main challenge in developing countries, especially in the least developed countries [123]. Orphan crops play an important role in global food and nutrition security for the livelihood of resource-poor farmers [124] and may have the potential to contribute to sustainable food systems under climate change [125]. Orphan crops like cowpea are indigenous and are invariably grown by small and marginal farmers under a subsistence farming system [124].

Cowpea is a future smart food with a high contribution to food and nutritional security and is good for medicinal purposes as well as being an option in extreme environments [88]. Among underutilized pulse crops, cowpea is the most nutritionally dense and climate-resilient, as it is amenable to diverse cropping systems and is locally available for economic growth and social development [122,125]. Cowpea is a multipurpose legume crop. It has high-quality protein and is rich in macro and micro-nutrients for human consumption. It is also rich in protein for livestock fodder and for human consumption. It improves soil fertility by recycling nutrients through nitrogen fixation in association with nodulating bacteria [86] and is weed suppressing [126,127]. This crop is more drought-tolerant than other pulse crops [4].

**Figure 2:**
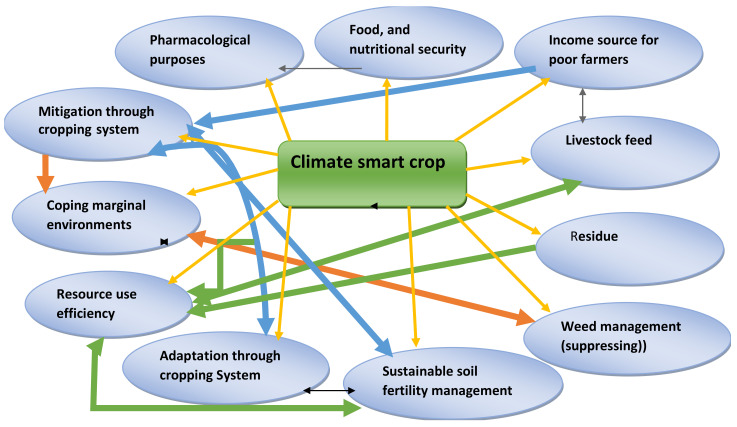
Cowpea as a climate-smart crop for SSA.

Cowpea as a vegetable contains important nutrients, including vitamins and minerals, that can improve the nutritional status of individuals and households with proper utilization [103]. The high nutritional value of cowpea leaves makes them ideal for efforts aimed at reducing food and nutrition insecurity. Cowpea can provide considerable amounts of bioavailable nutrients that are useful to alleviate nutrient deficiency among rural and urban populations. Plant-derived minerals and protein are the cheapest alternatives to circumvent the malnutrition that is prevalent in SSA [84]. In general, cowpea can be used as a climate change-resilient legume crop and as a future super crop in marginal environments (Figure 2).

## 6. Cowpea Genetics and Breeding Progress

Cowpea is an annual self-pollinated diploid (2n = 2x = 22), warm-season, multifunctional legume grown for food, fodder, vegetable, and green manure [127] with a 620 Mb genome size [128]. Different breeding methods for cowpea improvement have been widely and successfully used, including pure line selection, mass selection, pedigree, backcross, and single-seed descent [129]. Earliness, growth habit, resistance to biotic stress, drought tolerance, a high and stable yield, and good nutritional profile and quality are the most important traits for cowpea breeding [16,25,129]. For agronomic trait improvements in cowpea, pure line selection, mass selection, pedigree, backcross, and single seed descent have been used, utilizing additive, dominance, additive × additive, additive × dominance, and dominance × dominance effects different traits [130]. Several traits have been improved through conventional and molecular breeding tools to harness cowpea genetic variation for breeding [131].

### 6.1. Conventional Breeding

Leaf shape, leaf size, leaf number per plant, pod number per plant, and pod length are important characteristics that can be used for classifying and distinguishing cowpea varieties. However, few breeding methods have focused on the leaves compared to seeds. Leaf shape can also be used as a morphological or physical characteristic during the selection process if it is closely linked with an agronomic trait of interest. Narrow or hastate leaf shapes characterize most cowpea wild relatives, while cultivated genotypes have the ovate or sub-globose leaf shape. However, a possible adaptive advantage for narrow leaves in wild cowpea has not been investigated. The hastate leaf shape was shown to be dominant to the ovate leaf shape, which could be attributed to direct or indirect selection by breeders.

The genetic control of leaf shape is unknown. Other studies have reported that leaf shape is controlled by the number of cell cycles that occur during leaf development [132]. The leaf shape is influenced by environmental factors, including light intensity, temperature, and humidity [133]. The effect of genetic control and environmental factors on the development and the nature of leaf shape is unknown.

Understanding the variation among cowpea genotypes for leaf shape and size could potentially assist breeders in improving the crop for drought tolerance and yield. Previous studies [134] have shown that drought tolerance is associated with small leaf size, while other studies have reported that leaf shape and size are associated with photosynthesis rate and capacity. It has also been reported that leaf shape is not affected by geographic region [135], while other studies [136] have reported the opposite.

Several studies have investigated the variability in the nutritional value of cowpea leaves [59,60] in single experimental trials. Cowpea leaf protein values evaluated in different locations ranged from 25.1 to 43.1% [96,97]. This large variation could potentially be due to genetic and environmental effects. The iron concentration and β-carotene content in the same 561 samples ranged from 140.5 to 3994.7 µg/g and 4.1 to 30.5 mg/100 g, respectively. Contents of up to 18.7 mg of Fe, 0.547 mg of Zn, and 4.45 mg of beta carotene per 100 g of the edible portion of freeze-dried raw cowpea in three districts, Kongwa, Singida and Arumeru, in Tanzania have been reported [136]. In South Africa, mineral concentrations in cowpea leaves have been reported to be 142 to 626 mg·kg^−1^ for Fe, 49 to 104 mg/kg^−1^ Zn, 196 to 394 mg/kg^−1^ Mn, 8.6 to 19.7 mg/kg^−1^ Cu and 42 to 55 mg/kg^−1^ B [96]. These studies indicated sufficient variability among the tested cowpea genotypes for breeding for improved nutritional value. The above findings also suggested the need to comprehensively investigate the effect of genotype × environment (i.e., environments and seasons) interactions on cowpea genotypes for nutritional value.

Several studies have focused on investigating genotype × environment (G × E) interactions and the change in the ranking of cowpea genotypes for seed grain in Egypt [100], Brazil [133], Ethiopia [134], Zimbabwe [135], and South Africa [136,137,138]. Fewer studies [97] have focused on G × E for cowpea yield and nutritional values. Another study [97] investigated the variability of 25 genotypes for nutritional value in cowpea leaves in one location for two seasons. Thus, only the genotype × season effect was evaluated [138].

### 6.2. Molecular Breeding

Molecular tools and genomic resources have been developed for cowpea crop improvement [139,140,141,142,143,144,145]. These integrated genomic resources include a 1536 single nucleotide polymorphism (SNP) genotyping platform, and an EST-derived SNP consensus genetic map has been developed. Using the same SNP markers, a cowpea physical map has been partially anchored to the cowpea consensus genetic map. About 500 diverse cowpea accessions have been SNP-genotyped, and the initial cowpea genome has been assembled. These resources will enable the dissection of underlying genetic components of target agronomic traits using Quantitative Trait Locus (QTL) analysis and association mapping.

Numerous biparental populations have been used to map major QTLs for various traits [140,141,142] and to develop consensus genetic maps of cowpea [144,145]. For example, one study [146,147] focused on using the SNP markers to map the hastate versus ovate leaf shape trait in a biparental recombinant inbred line (RIL) population. In addition, new populations have been developed for higher-resolution mapping, including eight parents and 305 lines [140]. Recently, a reference genome sequence of cowpea [144] and genome assemblies of six additional diverse accessions [128] have been produced. The identified and confirmed QTLs would facilitate cultivar improvement using marker-assisted breeding.

#### 6.2.1. Marker–Trait Associations

Marker–trait association analysis has become a valuable tool in functional plant genomics and high-resolution mapping of QTL [148,149]. Several linkage maps have been used to identify QTLs for desirable traits in cowpea breeding (Table 5). In cowpea, understanding flowering time is a key player in plant adaptation and is an important phenological trait for breeding because agronomic traits such as plant growth, plant height, pod number, pod length, and nutritional quality traits depend on the time of flowering [150]. Previous studies have been focused on identifying QTL using SNP and simple sequence repeat (SSR) markers in RILs. SNP and SSR markers were utilized in another RIL population of ZN016 × ZJ282 to identify QTLs for days to flowering, nodes to first flower, leaf senescence, and pod number per plant [151].

#### 6.2.2. Transcriptomics

Transcriptomics (microarrays, RNA-seq) is the complete set of transcripts in a cell and their quantity for a specific developmental stage or physiological condition. Transcriptome analyses may help to understand how the transcriptome changes contribute to various cellular processes, gene discoveries, and putative gene functions [157,158,159,160,161]. In cowpea, some researchers have been using the transcriptomics approach. In IT97K-499-35, a total of 27cDNA libraries were generated from the major vegetative tissues (roots, stem, and flower of five-week-old plants) (http://vugea.noble.org accessed on 19 April 2022). Using the roots, stem, and leaves of cowpea seedlings of 32 cowpea accessions with the Illumina sequencing techniques, 54 million high-quality cDNA sequences were identified and assembled into 47,899 unigenes containing 5560 genic SSR markers [162,163]. These results showed that genic-SSRs are valuable genetic resources for use in various breeding strategies to increase the efficiency of cowpea improvement where molecular markers and genomic selection resources are limited.

Early developing seed tissues of two cowpea genotypes (IT86D-1010 and IT97K-499-35) using the Illumina HiSeq 2500 system generated 125 to 265 million reads, which were assembled into 35,000 to 74,000 transcript contigs [164] and were used to develop transcriptomic resources to characterize expressed genes in leaves and notably floral tissues undergoing male and female gametogenic development and early seed initiation. The majority of the transcript contigs across all the tissues in both genotypes could be mapped to the cowpea v1.0 reference genome. This is important for future exploration of cis-regulatory regions associated with tissue-specific gene expression. Distinct changes in global gene expression profiles that occur in host roots following successful and unsuccessful attempted parasitism by Striga may help to understand the resistance mechanism. The induction of specific defense-related genes and pathways defines the components of a unique resistance mechanism [165].

### 6.3. Genetic Resource Management

Crop genetic resources are an essential component of agrobiodiversity. The genetic materials of crops have value as a resource for present and future material developments for sustainable uses [166]. Cowpea germplasm resources are also strategic resources that are essential to national and global agricultural security through sustaining the desirable traits on the target crops for crop breeding, research, and conservation management and the long-term resiliency of food security. The International Institute of Tropical Agricultural (IITA) maintains the world’s largest collection of cowpea germplasm of over 15,003 accessions from 90 countries in its gene bank [167]. More than 80% of accessions were characterized for 28 agro-botanical descriptors of cowpea [167,168]. In addition to IITA, the United States Department of Agriculture-genetic Resources Information Network (USDA-GRIN) at Griffin, the USA and the University of California, Riverside, USA, are also conserving cowpea collection of about 7737 and 6000 accessions, respectively [169,170]. At this time, there is a larger war on seed ownership in the African content and globally [171]. A comprehensive collection, conservation, and cowpea characterization for each growing region will be crucial for future development of new varieties and identification of desirable genes for specific traits. The existing and future collections are sources of genes that are needed for enhancing the productivity of new, improved varieties in short, medium, and long-term strategies for climate change resilience.

## 7. Breeding Strategies and Research Perspectives for Cowpea

**Figure 3:**
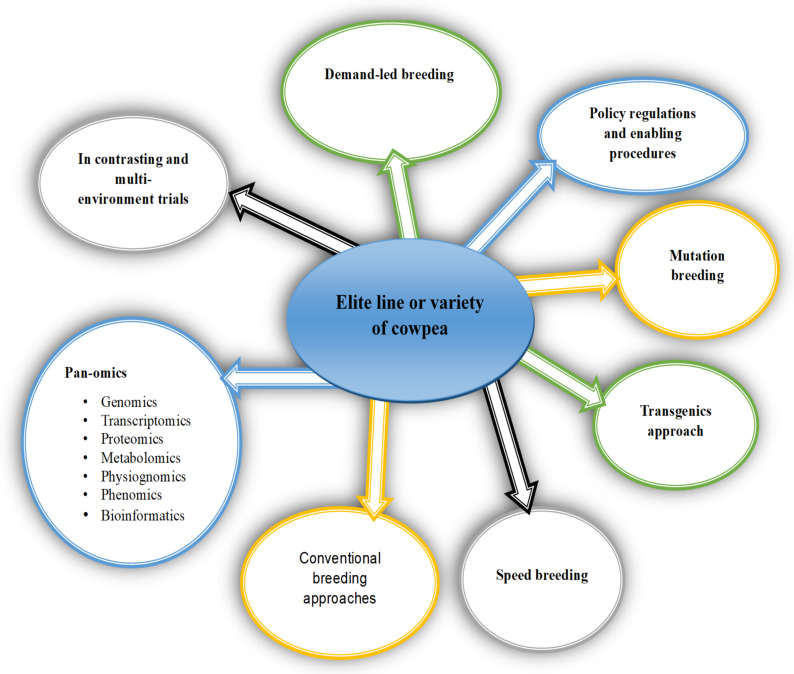
Breeding strategies and research perspectives of cowpea for food and nutritional security.

The application of modern genomic selection and agronomic tools to improve indigenous/orphan crops can provide enormous and novel opportunities for ensuring global food and nutritional security [172]. Developing desirable cowpea varieties for sustainable uses in tropical and sub-tropical regions would employ a wide range of conventional and contemporary breeding strategies (Figure 3). Implementing a structural breeding program that takes advantage of additional modern crop improvement tools such as genomic selection, speed breeding, genomic editing, mutation breeding, transgenic approaches, high throughput phenotyping, and breeding digitization would allow rapid improvements to orphan crops [173].

### 7.1. Genomic Selection

Genomic selection (GS) is a promising approach for exploiting DNA markers to design novel breeding programs and to develop new marker-based models for genetic evaluation and unlocking of traits [174]. In cowpea breeding as a vegetable, GS has opened new avenues to implement simultaneous selection for several traits [175], and provides opportunities to increase the genetic improvement of complex traits per unit of time and cost [175].

In this crop, genomic strategies have significantly accelerated the development of climate-smart genetic resources or elite vegetable varieties [131,132,176], through identifying genetic diversity and favorable variation required for climate resilience and food and nutritional security, identifying traits and genes for tolerance to new and complicated stresses induced by climate change and taking integrated genomics tools and approaches to manage combining tolerance to abiotic and biotic stresses as well as improved nutritional profiles and quality of grain, leaves and the immature pods of cowpea [177]. Increasing genetic gains and breeding efficiency through a rapid-cycle genome-wide selection could be a future cost-effective and precise method for orphan crop improvement for human food and animal feed and rehabilitation of poor soils of overexploited cultivated land [178].

### 7.2. Speed Breeding

The current world population is 7.8 billion and is projected to reach 9.7 billion by 2050 [179]. Climate fluctuations involving rising temperatures, more frequent floods, and frequent and prolonged drought episodes are predicted and could lead to frequent pest outbreaks and new disease pandemics with high severity. In addition, agricultural production should be increased by 60–110% to meet the global requirement of the projected population by 2050 [180]. Around 2 billion people are facing extreme micronutrient deficiencies, and more than 815 million are suffering from chronic hunger [181]. Therefore, tackling existing and upcoming challenges with a fast and effective response requires accelerating plant breeding as one of the best fitting approaches [182] and the key technologies that would revolutionize the breeding of orphan crops [183]. Speed breeding protocols could be applied to shorten breeding cycles and accelerate the improvement activities of orphan crops [183]. Essentially, speed breeding (SB), commonly known as fast-tracked breeding, is an additional tool available to speed up plant breeding [184]. SB is the deliberate manipulation of various growing conditions and has been used on various crops to rapidly develop iso-lines after initial crosses of target parents with complementary traits. The techniques depend on the manipulation of photoperiod, light intensity, temperature, soil moisture, soil nutrition, and high-density planting for developing an elite variety for immediate use [185]. SB, therefore, could be very promising when implemented in the right way to enhance underutilized crops [183].

### 7.3. Mutation Breeding

Climate change is rapidly changing how we live, what we eat, and how we eat and produce the underutilized crops we breed and the target traits [173]. For more than 70 years, mutation breeding has been used for crop improvement for different traits [186]. Mutation breeding has been used for improving both the oligogenic and polygenic traits of many crops. It has been employed to enhance morphological and physiological traits, biotic and abiotic stress resistance, yielding ability and nutritional quality, growth habits, and preferred end-user characteristics in crops for better trait selection and in order to contribute to global food security [186,187]. The application of numerous induced mutations was used to correct one or a few negative characters and to get new gene combinations which are desirable without changing the plant’s total genetic makeup [187]. In creating variability through mutagenesis, either physical (fast neutron, UV, X-ray, and gamma radiation) or chemical (N-methyl-N-nitrosourea (MNU), sodium azide, hydrogen fluoride (HF), methyl methanesulfonate (MMS), or ethyl methanesulfonate (EMS) have been widely used for new material development [188] and are important for developing improved varieties in the field of functional genomics [141,144]. In addition, Agrobacterium and transposon-based chromosol integration for complex traits like yield are important [189]. Globally, over 3000 new varieties have been released using mutation breeding, including some neglected crops in Africa [129]. Mutation breeding helps to enhance the tolerance of crops to diverse climatic conditions such as high temperatures, drought, and the occurrence of insect pests and diseases [141,145].

### 7.4. Genome Editing

Genome or gene editing provides precise, heritable genome mutagenesis without permanent transgenesis, and has been widely demonstrated in crops using a conventional method that is applied to alter the genotype and phenotype of crops and comprises the use of both natural and artificial crosses and induced mutations [190] and modern genome editing [191] approaches for crop improvement. Currently, genome editing (GE) could be used to rapidly modify undesirable traits in neglected crops to accelerate the process of domestication and for sustaining food and nutritional security [192].

Compared to the well-studied crops, minor crops have limiting factors, including a lack of annotated genomes, sub-optimal tissue culture regeneration protocols and a lack of genetic transformation methods [183]. Therefore, to fill these existing GE knowledge gaps, a knowledge model or the study of other crops or more closely related species can be used to translate genetic knowledge from one crop to another [193]. Thus, as more genome sequences become available, it becomes easier to understand and use the information for crops currently being domesticated and to identify orthologs of known domestication genes [192]. In recent years, rapid modern GE technologies, such as zinc finger nuclease (ZFNs), transcription activator-like nucleases (TALENs), and cluster regularly interspaced short palindromic repeats/CRISPR associated protein (CRISPR/Cas), meganucleases and targeted regularly double-strand breaks (DSBs) have been developed [193], which demonstrates the broad applications used in the discovery of new genes or undesirable gene functions [192].

## 8. Policy and Regulatory Framework

A country’s national policies have a direct impact on the adoption and expansion of cowpea as a climate-smart crop, and without support from local leaders and policymakers, use of this crop as a food security crop is limited. Many SSA countries are still lacking in the capacity and essential procedures required to establish adequate policies, regulations, implementation, and monitoring frameworks regarding seed systems (including the whole value chain) [194,195] and the development and production of multi-nutrient purposes of cowpea. In SSA countries, the major bottleneck to the adoption of the genetic modified organism (GMOs) and deployment of Bt-expressing cowpea in Africa is the lack of political will and of external influence. Leaders and policymakers should be able to make informed decisions and publicly defend such decisions on GM technology. Therefore, the involvement of scientists, industrial research councils, and policymakers should work together to create awareness and advocate policy changes to permit GM technology.

## 9. Conclusions

Cowpea is a future smart food crop with excellent nutritional and nutraceutical properties. It has several agronomic, environmental, and economic advantages in underdeveloped and developing countries, contributing to food and nutritional security. Cowpea is truly a multifunctional climate-resilient crop for promoting global food security and maintaining good health to reduce non-communicable human diseases. In addition, cowpea leaves and immature pods can be used as food by millions of people in the developing world and can be used as a food ingredient in the food industry.

However, biotic (viral, fungal, and bacterial diseases, nematodes, aphids, parasitic weeds, and several insect pests) and abiotic stress (such as phosphorus stress in the soil; heat and drought stress), cultural beliefs, low yield, limited attention from researchers, limited developments, as well as policymakers and limited modern breeding technology resources for cowpea breeding as a vegetable crop, are major challenges for this neglected crop.

Nutritionally rich cowpea vegetable cultivars can be developed using existing genetic resources for target traits, and with the integration of chance breeding (mutation, hybridization, backcrossing, pedigree, and recurrent selection), modern breeding approaches (space breeding, speed breeding, genomic selection, and genome editing), technological innovation (mutagenesis breeding), transgenic approaches, demand-led breeding and multi-omics approaches would be the best solution for tackling the existing production problem in SSA.

With the use of all possible breeding methods and approaches, future cowpea crop improvements should focus on breeding for high yielding cultivars with good nutritional quality traits and resistance to biotic stresses and tolerance to abiotic stresses. High yielding cultivars with a good nutritional value, as well as resistance and tolerance to diseases and pests, could potentially be used as parents for future crop improvement, which will result in the development of superior varieties. Successful breeding for superior cowpea genotypes is expected to contribute to food security and to help combat malnutrition in Africa and beyond.

## Figures and Tables

**Table 1:** Amino acid composition of cowpea grain and leaves.

Amino Acid	Leaves (g/100 g Protein)		Grain (g/100 g Protein)	
	Mean Range	References	Mean Range	References
Aspartic acid	10.8–26.7	[86,88]	6.0–13	[86,89,90]
Arginine	7.4–17.3	[88,91]	5.0–10.8	[86,90]
Alanine	4.2–9.8	[91,92,93]	3.4–5.1	[10]
Methionine	1.0–4.5	[91,94]	0.9–3.5	[91,94]
Glutamic acid	17.2–45.3	[91,93]	8.5–19	[91,95]
Glycine	3.8–12.6	[91,93]	3.1–4.8	[94,96]
Cysteine	0.5–2.9	[86,93]	0.3–2.4	[91,96]
Histidine	1.8–8.6	[91,94]	2.0–4.41	[94,97]
Isoleucine	4.1–11.1	[84,91]	2.8–5.4	[94,97]
Leucine	7.4–19.6	[91,94]	5.7–11.3	[94,97]
Lysine	3.0–16.3	[91,94]	3.5–8.0	[5]
Phenylalanine	4.6–14.4	[91,94]	4.4–9.9	[94,95]
Proline	4.0–15.9	[91,93]	3.1–8.9	[91,95]
Serine	3.0–11.6	[91,93]	3.8–5.8	[94,95]
Threonine	3.2–10.8	[84,91]	3.0–5.9	[94,97]
Tryptophan	1.3–4.1	[91,93]	0.9–1.5	[94,95]
Tyrosine	3.0–9.3	[91,93]	2.6–4.5	[5]
Valine	5.0–12.8	[91,93]	3.4–6.2	[91,94]

**Table 2:** Mineral composition of cowpea grain, immature pods, and leaves.

	Leaves		Immature Pod		Grain	
Minerals	Mean Range	References	Mean Range	References	Mean Range	References
Macro-minerals (mg/100 g dry matter)						
Calcium	15.2–46.20	[15]	223.67–867.77	[16]	0.07–2.7	[89,94]
Phosphorus	2.3–6.10	[15]	383.43–537.53	[16]	2.1–592.4	[89,94]
Potassium	9.30–35.60	[15]	170.74–240.78	[10,85]	9.57–1445.2	[89,95]
Magnesium	4.3–8.4	[15]	297.97–426.20	[16]	1.3–227.4	[89,99,100,101]
Sulfur	153.3–200.0	[24]			120.0–147.3	[42]
Micro-minerals (mg/100 g dry matter)						
Copper	0.15–2.2	[101,102,103]	0.48–0.95	[16]	0.5–2.2	[95,101]
Iron	26.76–182.33	[25]	6.01–9.78	[16]	3.4–10.6	[89,104]
Manganese	10.57–204	[101,103]	2.11–4.77	[16]	1.38–4.3	[89,101]
Sodium	11.59–43.95	[25]	13.70–32.93	[16]	8.4–79.81	[89,95]
Zinc	2.78–22.3	[101,103]	1.42–5.63	[10,85]	2.4–5.11	[89,94]
Aluminum			1.84–7.86	[16]		
Boron	3.14–5.01	[27]	2.13–4.03	[16]	1.47–2.14	[27]
Selenium			2.5–3.4	[23]		

**Table 3:** List of vitamins in cowpea grain.

Vitamins	Mean Range/Mean (%)	References
Vitamin A	0.00–0.07	[5]
Vitamin B1	0.2–1.7	[5]
Vitamin B2	0.1–76	[91,107]
Vitamin B3	0.7–4.0	[5]
Vitamin B5	1.7–2.2	[5]
Vitamin B6	0.2–0.41	[5]
Vitamin B7	0.02–0.03	[5]
Vitamin B9	0.1–0.4	[5]
Vitamin B12	0 or trace	[5]
Vitamin C	1.5–1.69	[28]
Vitamin D	0.00	[28]
Vitamin E	0.07–20	[5]

**Table 4:** Proximate and fiber composition of leaves and grain.

Nutrient	Leaves		Grain	
	Mean Range (%)	References	Mean Range (%)	References
Moisture	8–9	[30]	11.81–13.24	[31]
Ash	8.1–14.4	[10]	3.1–5.8	[10]
Crude protein	27–43	[32]	21–33	[32]
Crude lipid	1.3–4.1	[10]	0.5–3.9	[10]
Crude fiber	10.09–35.9	[94,108,109]	18–32	[11]
Carbohydrate	59.7–65.2	[33]	50–60	[34]

**Table 5:** Breeding achievements of cowpea using marker–trait association.

Traits	Population	Type	Marker Type	QTLs	References
Cowpea golden mosaic virus	IT97 K-499-35 × Canapu T16	F2	AFLP	3	[147]
Fusarium wilt resistance	CB27 × 24–125B-1	RIL	SNP	1	[148]
Days to flowering	524B × 219-01	RIL	SSR	3	[152]
Pod length	(JP81610 × TVNU457) × JP81610	BC1F1	SSR	9	[153]
Pod tenderness	(JP81610 × JP89083) × JP81610	BC1F1	SSR	3	[154]
Foliar thrips	CB46 × IT93 K-503-1 and CB27 × IT82E-18	RILs	SNP	3	[155]
Cowpea bacterial blight resistance	Danlla × TVu7778	RIL	SNP	3	[155]
Charcoal rot resistance	IT93 K-503-1 × CB46	RIL	SNP	9	[150]
Striga gesnerioides	TVx 3236 × IT82D-849	F2	AFLP	3	[151]
Hastate leaf shape	Sanzi × Vita 7	RIL	SNP	1	[148]
Pod fiber layer thickness	524B × 219-01	RIL	SSR	4	[156]
Pod number per plant	ZN016 × ZJ282	RIL	SSR	3	[146]
Pod tenderness	(JP81610 × JP89083) × JP81610	BC1F1	SSR	3	[154]
Nodes to the first flower	ZN016 × ZJ282	RIL	SNP	4	[146]
Days to first flowering	ZN016 × ZJ282	RIL	SNP	3	[146]
Days to maturity	IT93K503-1 × CB46	RIL	AFLP	2	[155]

## Data Availability

Not applicable.

## References

[1] Gonçalves A., Goufo P., Barros A., Domínguez-Perles R., Trindade H., Rosa E.A., Ferreira L., Rodrigues M. (2016). Cowpea [*Vigna unguiculata* (L.) Walp] a renewed multipurpose crop for a more sustainable agri-food system: Nutritional advantages and constraints. J. Sci. Food Agric..

[2] Carvalho M., Lino-Neto T., Rosa E., Carnide V. (2017). Cowpea: A legume crop for a challenging environment. J. Sci. Food Agric..

[3] Halilou O., Hamidou F., Taya B.K., Mahamane S., Vadez V. (2015). Water use, transpiration efficiency and yield in cowpea (*Vigna unguiculata*) and peanut (*Arachis hypogaea*) across water regimes. Crop Pasture Sci..

[4] Timko B.B., Singh M.P., Paul M., Moore H., Ray M. (2008). Cowpea, a multifunctional legume. Genomics of Tropical Crop Plants.

[5] Sikora R.A., Claudius-Cole B., Coyne E.J., Sikora R.A., Coyne D., Hallmann J., Timper P. (2018). Nematode parasites of food legumes. Plant Parasitic Nematodes in Subtropical and Tropical Agriculture.

[6] Jayathilake C., Visvanathan R., Deen A., Bangamuwage R., Jayawardana B.C., Nammi S., Liyanage R. (2018). Cowpea: An overview on its nutritional facts and health benefits Running title: Nutritional and health properties of cowpea. J. Sci. Food Agric..

[7] Deo-Donne P.L., Annan S.T., Adarkwah F., Pady F., Anyamesem-Poku A. (2018). Reaction of Cowpea genotypes to bacterial blight (*Xanthomonas campestris* pv. *Vignicola*) disease in Ghana. World J. Agric. Res..

[8] De La Peña T.C., Pueyo J.J. (2012). Legumes in the reclamation of marginal soils, from cultivar and inoculant selection to transgenic approaches. Agron. Sustain. Dev..

[9] De Ron A.M., Prohens-Tomás J., Nuez F., Carena M.J. (2015). Grain legumes. Handbook of Plant Breeding.

[10] Gerrano A.S., van Rensburg W.S.J., Adebola P.O. (2017). Nutritional composition of immature pods in selected Cowpea [*Vigna unguiculata* (L.) Walp.] genotypes in South Africa. Aust. J. Crop Sci..

[11] UNICEF/WHO/World Bank (2021). Levels and Trends in Child Malnutrition UNICEF/WHO/World Bank Group Joint Child Malnutrition Estimates Key Findings of the 2021 Edition. https://www.who.int/publications/i/item/9789240025257.

[12] Global Panel on Agriculture Food Systems for Nutrition (2020). Future Food Systems: For People, Our Planet, and Prosperity.

[13] May J., Witten C., Lori L. (2020). Challenges and Opportunities for Water, Sanitation, Hygiene (WASH) and Infant Nutrition in South Africa.

[14] National Department of Health (NDoH), Statistics South Africa (Stats SA), South African Medical Research Council (SAMRC), ICF (2019). South Africa Demographic and Health Survey 2016.

[15] Statistics South Africa (2017). Poverty Trends in South Africa; An Exammination of Absolute Poverty between 2006 and 2015.

[16] Thow A.M., Greenberg S., Hara M., Friel S., Sanders D. (2018). Improving policy coherence for food security and nutrition in South Africa: A qualitative policy analysis. Food Secur..

[17] Mohammed S.B., Dzidzienyo D.K., Umar M.L., Ishiyaku M.F., Tongoona P.B., Gracen V. (2021). Appraisal of cowpea cropping systems and farmers’ perceptions of production constraints and preferences in the dry savannah areas of Nigeria. CABI Agric. Biosci..

[18] Nedumaran S., Abinaya P., Jyosthnaa P., Shraavya B., Rao P., Bantilan C. (2015). Grain Legumes Production, Consumption and Trade Trends in Developing Countries.

[19] FAOSTAT—Food and Agriculture Organization of the United Nations (2022). Statistical Databases.

[20] Kebede E., Bekeko Z. (2020). Expounding the production and importance of cowpea [*Vigna unguiculata* (L.) Walp.] in Ethiopia. Cogent Food Agric..

[21] Boukar O., Belko N., Chamarthi S., Togola A., Batieno J., Owusu E., Haruna M., Diallo S., Umar L.M., Olufajo O. (2019). Cowpea (*Vigna unguiculata*): Genetics, genomics and breeding. Plant Breed..

[22] Enyiukwu D., Amadioha A., Ononuju C. (2018). Nutritional Significance of cowpea leaves for human consumption. Greener Trends Food Sci. Nutr..

[23] Kukal M.S., Irmak S. (2018). Climate driven crop yield and yield variability and climate change impacts on the U.S. great plains agricultural production. Sci. Rep..

[24] FAO (2015). Climate Change and Food Security: Risks and Responses.

[25] Ganiyu S.A., Popoola A.R., Owolade O.F., Fatona K.A. (2017). Control of common bacterial blight disease of cowpea (*Vigna unguiculata* [L.] Walp) with certain plant extracts in Abeokuta, Nigeria. J. Crop Improv..

[26] Agbicodo E.M., Fatokun C.A., Bandyopadhyay R., Wydra K., Diop N.N., Muchero W., Ehlers J.D., Roberts P.A., Close T.J., Visser R.G.F. (2010). Identification of markers associated with bacterial blight resistance loci in cowpea [*Vigna unguiculata* (L.) Walp.]. Euphytica.

[27] Adegbite A.A., Amusa N.A. (2010). The major economic field diseases of cowpea in the humid agro-ecologies of south-western Nigeria. Arch. Phytopathol. Plant Prot..

[28] Taiwo M.A., Akinjogunla O.J. (2006). Cowpea viruses: Quantitative and qualitative effects of single and mixed viral infections. Afr. J. Biotechnol..

[29] Salem N.M., Ehlers J.D., Roberts P.A., Ng J.C.K. (2010). Biological and molecular diagnosis of seedborne viruses in cowpea germplasm of geographically diverse sub-Saharan origins. Plant Pathol..

[30] Ogunsola K.E., Ilori C., Fatokun C.A., Boukar O., Ogunsanya P., Kumar P.L. (2021). Disease incidence and severity in cowpea lines evaluated for resistance to single and multiple infections of endemic viruses in Nigeria. J. Crop Improv..

[31] Obopile M., Ositile B. (2010). Life table and population parameters of cowpea aphid, Aphis craccivora Koch (Homoptera: Aphididae) on five cowpea [*Vigna unguiculata* (L.) Walp.] varieties. J. Pest Sci..

[32] Oliveira J.T., Andrade N.C., Martins-Miranda A.S., Soares A.A., Gondim D.M., Araújo-Filho J.H., Freire-Filho F.R., Vasconcelos I.M. (2012). Differential expression of antioxidant enzymes and PR-proteins incompatible and incompatible interactions of cowpea (*Vigna unguiculata*) and the root-knot nematode Meloidogyne incognita. Plant Physiol. Biochem..

[33] Kamara A.Y., Ekeleme F., Jibrin J.M., Tarawali G., Tofa I. (2014). Assessment of level, extent and factors influencing Striga infestation of cereals and cowpea in a Sudan Savanna ecology of northern Nigeria. Agric. Ecosyst. Environ..

[34] Omoigui L.O., Kamara A.Y., Alunyo G.I., Bello L.L., Oluoch M., Timko M.P., Boukar O. (2017). Identification of new sources of resistance to Striga gesnerioides in cowpea (*Vigna unguiculata*) accessions. Genet. Resour. Crop Evol..

[35] Ohanmu E.O., Ikhajiagbe B. (2018). Competitive effect of prominent weeds on cowpea cultivar in a typical ultisol. Am. J. Plant Physiol..

[36] Razakou A., Ibrahim M., Adamou I., Karimou A., Mahamane S., Adam T., Moumouni S. (2017). Evaluation of cowpea lines on natural infested field of Striga gesnerioides in the Sahel Sudan of Southeast Niger. Agric. Sci. Res. J..

[37] Gupta K.C., Gupta A.K., Saxena R. (2016). Weed management in cowpea [*Vigna unguiculata* (L.) Wasp.] under rainfed conditions. Int. J. Agric. Sci..

[38] Prabhu G., Srinivasan R., Kantwa S.R., Palsaniya D.R., Chaudhary M. (2015). Weed seed bank studies in the field of fodder cowpea [*Vigna unguiculata* (L.)]. Int. J. Appl. Pure Sci. Agric..

[39] Marinov-Serafimov P. (2010). Determination of allelopathic effect of some invasive weed species on germination and initial development of grain legume crops. Pestic. Fitomed..

[40] Fisichellaelli N.A., Abella S.R., Peters M., Krist F.J. (2014). Climate, trees, pests, and weeds: Change, uncertainty, and biotic stressors in eastern U.S. national park forests. For. Ecol. Manag..

[41] Nisha T.Y., Chopra K., Chopra N.K., Yadav M.R., Kumar R., Rathore D.K., Soni P.G., Makarana G., Tamta A., Kushwah M. (2017). Weed Management in Cowpea-A Review. Int. J. Curr. Microbiol. Appl. Sci..

[42] Egho E. (2011). Management of major field insect pests and yield of cowpea [*Vigna unguiculata* (L) Walp] under calendar and monitored application of synthetic chemicals in Asaba, southern Nigeria. Am. J. Sci. Ind. Res..

[43] Adelaïde P.O., Batieno B.J., Traore F., Jean-Baptiste T., Huynh B., Roberts P.A., Close T., Ouédraogo J.T. (2018). Screening of cowpea [*Vigna unguiculata* (L.) Walp.] lines for resistance to three Aphids (Aphis craccivora Koch) strains in Burkina Faso. Afr. J. Agric. Res..

[44] Souleymane A., Aken’Ova M.E., Fatokun C.A., Alabi O.Y. (2013). Screening for resistance to cowpea aphid (*Aphis craccivora* Koch) in wild and cultivated cowpea [*Vigna unguiculata* (L.) Walp.] accessions. Int. J. Sci. Environ. Technol..

[45] Togola A., Boukar O., Servent A., Chamarthi S., Tamò M., Fatokun C. (2020). Identification of sources of resistance in cowpea mini core accessions to Aphis craccivora Koch (Homoptera: Aphididae) and their biochemical characterization. Euphytica.

[46] Mofokeng M.A., Gerrano A.S. (2021). Efforts in breeding cowpea for aphid resistance: A review. Acta Agric. Scand. Sect. B Soil Plant Sci..

[47] Stoddard F.L., Nicholas A.H., Rubiales D., Thomas J., Villegas-Fernández A.M. (2010). Integrated pest management in faba bean. Field Crop. Res..

[48] Dugje I.Y., Omoigui L.O., Ekeleme F., Kamara A.Y., Ajeigbe H. (2009). Farmers’ Guide to Cowpea Production in West Africa.

[49] Anjum S.A., Xie X., Wang L., Saleem M.F., Man C., Lei W. (2011). Morphological, physiological and biochemical responses of plants to drought stress. Afr. J. Agric. Res..

[50] Chand U., Harsh J., Rintu N., Pronob J. (2020). Heat stress and cowpea: Genetics, breeding and modern tools for improving genetic gains. Plant Physiol. Rep..

[51] Chaudhary S., Devi P., Bhardwaj A., Jha U.C., Sperotto R.A., Kean D., Tan Y. (2020). Identification and characterization of contrasting genotypes/cultivars for developing heat tolerance in agricultural crops: Current status and prospects. Front. Plant Sci..

[52] Daryanto S., Wang L., Jacinthe P.A. (2017). Global synthesis of drought effects on cereal, legume, tuber and root crops production: A review. Agric. Water Manag..

[53] Fatokun C.A., Boukar O., Muranaka S. (2012). Evaluation of cowpea (*Vigna unguiculata* (L.) Walp.) germplasm lines for tolerance to drought. Plant Genet. Resour..

[54] Ghonaim M.M., Mohamed H.I., Omran A.A.A. (2021). Evaluation of wheat (*Triticum aestivum* L.) salt stress tolerance using physiological parameters and retrotransposon-based markers. Genet. Resour. Crop Evol..

[55] Jemo M., Sulieman S., Bekkaoui F., Olomide O.A.K., Hashem A., Abd Allah E.F., Alqarawi A.A., Tran L.S.P. (2017). Comparative analysis of the combined effects of different water and phosphate levels on growth and biological nitrogen fixation of nine cowpea varieties. Front. Plant Sci. Plant Sci..

[56] Korte F., Coulston F., Parlar P.H. (2018). Mitigation the harmful effect of salt stress on physiological, biochemical and anatomical traits by foliar spray with trehalose on wheat cultivars. Fresenius Environ. Bull..

[57] Krasilnikoff G., Gahoonia T., Nielsen N.E. (2003). Variation in phosphorus uptake efficiency by genotypes of cowpea (*Vigna unguiculata*) due to differences in root and root hair length and induced rhizosphere processes. Plant Soil..

[58] Kumar S., Thakur P., Kaushal N., Malik J.A., Gaur P. (2013). Effect of varying high temperatures during reproductive growth on reproductive function, oxidative stress and seed yield in chickpea genotypes differing in heat sensitivity. Arch. Agron. Soil Sci..

[59] Li B., Gao K., Ren H., Tang W. (2018). Molecular mechanisms governing plant responses to high temperatures. J. Integr. Plant Biol..

[60] Liu Y., Li J., Zhu Y., Jones A., Rose R.J., Song Y. (2019). Heat Stress in Legume Seed Setting: Effects, Causes, and Future Prospects. Front. Plant Sci..

[61] Munns R. (2002). Comparative physiology of salt and water stress. Plant Cell Environ..

[62] Namakka A., Jibrin D.M., Hamma I.L. (2017). Effect of phosphorus levels on growth and yield of cowpea (*Vigna unguiculata* (L.) Walp.) in Zaria, Nigeria. J. Dryl. Agric..

[63] Nkomo G.V., Sedibe M.M., Mofokeng M.A. (2021). Production constraints and improvement strategies of Cowpea (*Vigna unguiculata* L. Walp.) genotypes for drought tolerance. Int. J. Agron..

[64] Nunes C., Moreira R., Pais I., Semedo J., Simões F., Veloso M.M., Scotti-Campos P. (2022). Cowpea Physiological Responses to Terminal Drought—Comparison between Four Landraces and a Commercial Variety. Plants.

[65] Olajide A.A., Ilori C.O. (2017). Genetic variability, performance and yield potentials of ten varieties of cowpea (*Vigna unguiculata* (L) Walp) under drought stress. Legume Genom. Genet..

[66] Olaleye O., Olajire F., Robert A.C., Nnenna I. (2011). Phosphorus response efficiency in cowpea genotypes. J. Agric. Sci..

[67] Priya M., Siddique K.H.M., Dhankhar O.P., Prasad P.V., Rao B.H., Nair R.M., Nayyar H. (2018). Molecular breeding approaches involving physiological and reproductive traits for heat tolerance in food crops. Indian J. Plant Physiol..

[68] Ravelombola W. (2019). A Simple and Cost-effective Approach for Salt Tolerance Evaluation in Cowpea (*Vigna unguiculata*) Seedlings. Hortscience.

[69] Ravelombola W., Shi A., Weng Y., Mou B. (2018). Association analysis of salt tolerance in cowpea (*Vigna unguiculata* (L.) Walp) at germination and seedling stages. Theor. Appl. Genet..

[70] Sehgal A., Sita K., Kumar J., Kumar S., Singh S. (2017). Effects of drought, heat and their interaction on the growth, yield and photosynthetic function of lentil (*Lens culinaris* Medikus) genotypes varying in heat and drought sensitivity. Front. Plant Sci..

[71] Singh A., Baoule A.L., Ahmed H.G., Dikko A.U., Aliyu U., Sokoto M.B., Alhassan J., Musa M., Haliru B. (2011). Influence of phosphorus on the performance of cowpea (*Vigna unguiculata* (L) Walp.) varieties in the Sudan savanna of Nigeria. Agric. Sci..

[72] Sudharani Y., Mohapatra P.P., Pattanaik M., Hans H., Maitra S. (2020). Effect of Phosphorus on Cowpea (*Vigna unguiculata* L. Walp): A review. J. Pharmacogn. Phytochem..

[73] Verbree D.A., Singh B.B., Payne W.A. (2015). Genetics and heritability of shoot drought tolerance in cowpea seedlings. Crop Sci..

[74] Tharanathan R.N., Mahadevamma S. (2003). Grain legumes—A boon to human nutrition. Trends Food Sci. Technol..

[75] Adebooye O.C., Singh V. (2007). Tannins, phytate, amino acid, fatty acid and mineral nutrients of whole-grain and decorticated. J. Food Qual..

[76] Affrifah N.S., Phillips R.D., Saalia F.K. (2021). Cowpeas: Nutritional profile, processing methods and products—A review. Legume Sci..

[77] Mumuni A., Shaibu S.S., Mohammed H., Adams M.M., Stephen K.A. (2016). Farmer participatory pest management evaluations and variety selection in diagnostic farmer field Fora in cowpea in Ghana. Afr. J. Agric. Res..

[78] Kamara A.Y., Ewansiha S.U., Ajeigbe H.A., Okechukwu R., Tefera H., Boukar O., Omoigui L.O. Improvements in grain and fodder yield of cowpea (*Vigna unguiculata*) varieties developed in the Sudan savannas of Nigeria over the past four decades. Proceedings of the Fifth World Cowpea Conference.

[79] Omomowo O.I., Babalola O.O. (2021). Constraints and prospects of improving cowpea productivity to ensure food, nutritional security and environmental sustainability. Front. Plant Sci..

[80] Gerrano A.S., van Rensburg W.S.J., Ventera S.L., Shargie N.G., Amelework B.A., Shimelis H.A., Labuschagne M.T. (2019). Selection of cowpea genotypes based on grain mineral and total protein content. Acta Agric. Scand. Sect. B Soil Plant Sci..

[81] Elhardallou S.B., Khalid I., Gobouri A., Abdel-Hafez S. (2015). Amino acid composition of cowpea [*Vigna ungiculata* (L.) Walp] flour and its protein isolates. Food Nutr. Sci..

[82] Petchiammal C., Hopper W. (2014). Antioxidant activity of proteins from fifteen varieties of legume seeds commonly consumed in India. Int. J. Pharm. Pharm. Sci..

[83] Iqbal A., Khalil I.A., Ateeq N., Khan M.S. (2006). Nutritional quality of important food legumes. Food Chem..

[84] OECD (2020). Safety assessment of foods and feeds derived from transgenic crops; common bean, rice, cowpea and apple compositional considerations. Novel Food and Feed Safety.

[85] Carvalho A.F.U., Sousa N.M., Farias D.F., da Rocha-Bezerra L.C.B., da Silva R.M.P., Viana M.P., Gouveia S.T., Sampaio S.S., de Sousa M.B., de Lima G.P.G. (2012). Nutritional ranking of 30 Brazilian genotypes of cowpeas including determination of antioxidant capacity and vitamins. J. Food Compos. Anal..

[86] Khattab R.Y., Arntfield S.D., Nyachoti C.M. (2009). Nutritional quality of legume seeds as affected by some physical treatments. LWT—Food Sci. Technol..

[87] Vasconcelos I.M., Maia F.M.M., Farias D.F., Campello C.C., Carvalho A.F.U., Moreira R.A., Oliveira J.T.A. (2010). Protein fractions, amino acid composition and antinutritional constituents of high-yielding cowpea cultivars. J. Food Compos. Anal..

[88] Dakora F.D., Belane A.K. (2019). Evaluation of protein and micronutrient levels in edible cowpea [*Vigna unguiculata* (L.) Walp.] leaves and seeds. Front. Sustain. Food Syst..

[89] Forrest H.N. (2014). Handbook of Nutrition and Food.

[90] Belane A.K., Dakora F.D. (2012). Elevated concentrations of dietarily-important [*Vigna unguiculata* (L.) Walp.] genotypes: Implications for human nutrition and health. Food Nutr. Sci..

[91] Belane A.K., Dakora F.D. (2011). Levels of nutritionally-important trace elements and macronutrients in edible leaves and grain of 27 nodulated cowpea [*Vigna unguiculata* (L.) Walp.] genotypes grown in the Upper West Region of Ghana. Food Chem..

[92] Gerrano A.S., Adebola P.O., van Rensburg W.S.J., Venter S.L. (2015). Genetic variability and heritability estimates of nutritional composition in the leaves of selected cowpea genotypes [*Vigna unguiculata* (L.) Walp.]. HortScience.

[93] Boukar O., Massawe F., Muranaka S., Franco J., Maziya-Dixon B., Singh B., Fatokun C. (2011). Evaluation of cowpea germplasm lines for protein and mineral concentrations in grains. Plant Genet. Resour. Charact. Util..

[94] Asare A.T., Agbemafle R., Adukpo G.E., Diabor E., Adamtey K.A. (2013). Assessment of functional properties and nutritional composition of some cowpea (*Vigna unguiculata* L.) genotypes in Ghana. ARPN J. Agric. Biol. Sci..

[95] Devi C.B., Kushwaha A., Kumar A. (2015). Sprouting characteristics and associated changes in nutritional composition of cowpea (*Vigna unguiculata*). J. Food Sci. Technol..

[96] USDA (2021). Agricultural Research Service Food Data Central: Foundation Foods.

[97] Antova G.A., Stoilova T.D., Ivanova M.M. (2014). Proximate and lipid composition of cowpea (*Vigna unguiculata* L.) cultivated in Bulgaria. J. Food Compos. Anal..

[98] Owade J.O., Abong G., Okoth M., Mwang’ombe A.W. (2020). A review of the contribution of cowpea leaves to food and nutrition security in East Africa. Food Sci. Nutr..

[99] Khalid I.I., Elharadallou S.B. (2013). Functional properties of cowpea [*Vigna ungiculata* (L.) Walp] and Lupin (*Lupinus termis*) Flour and Protein Isolates. J. Nutr. Food Sci..

[100] Chikwendu J.N., Igbatim A.C., Obizoba I.C. (2014). Chemical composition of processed cowpea tender leaves and husks. Int. J. Sci. Res. Publ..

[101] Baptista A., Pinho O., Pinto E., Casal S., Mota C., Ferreira L.V.O. (2017). Characterization of protein and fat composition of seeds from common beans (*Phaseolus vulgaris* L.), cowpea [*Vigna unguiculata* (L.) Walp] and bambara groundnuts [*Vigna subterranea* (L.) Verdc] from Mozambique. J. Food Meas. Charact..

[102] Kapravelou G., Martínez R., Andrade A.M., Chaves C.L., López-Jurado M., Aranda P., Arrebola F., Cañizares F.J., Galisteo M., Porres J.M. (2015). Improvement of the antioxidant and hypolipidaemic effects of cowpea flours (*Vigna unguiculata*) by fermentation: Results of in vitro and in vivo experiments. J. Sci. Food Agric..

[103] Bello S.K., Yusuf A.A., Cargele M. (2018). Performance of cowpea as influenced by native strain of rhizobia, lime and phosphorus in Samaru, Nigeria. Symbiosis.

[104] Xu B., Chang S.K.C. (2012). Comparative study on antiproliferation properties and cellular antioxidant activities of commonly consumed food legumes against nine human cancer cell lines. Food Chem..

[105] Xiong S., Yao X., Li A. (2013). Antioxidant properties of peptide from cowpea seed. Int. J. Food Prop..

[106] Santos C.A.F., Boiteux L.S. (2013). Breeding biofortifed cowpea lines for semi-arid tropical areas by combining higher seed protein and mineral levels. Genet. Mol. Res..

[107] Duranti M. (2006). Grain legume proteins and nutraceutical properties. Fitoterapia.

[108] Uruakp F. (2015). Influence of cowpea (*Vigna unguiculata*) peptides on insulin resistance. J. Nutr. Health Food Sci..

[109] Apea-Bah F.B., Minnaar A., Bester M.J., Duodu K.G. (2014). Does a sorghum-cowpea composite porridge hold promise for contributing to alleviating oxidative stress?. Food Chem..

[110] Ojwang L.O., Banerjee N., Noratto G.D., Angel-Morales G., Hachibamba T., Awika J.M., Mertens-Talcott S.U. (2015). Polyphenolic extracts from cowpea (*Vigna unguiculata*) protect colonic myofibroblasts (CCD18Co cells) from lipopolysaccharide (LPS)-induced inflammation-modulation of microRNA 126. Food Funct..

[111] Frota K.M.G., Mendonça S., Saldiva P.H.N., Cruz R.J., Arêas J.A.G. (2008). Cholesterol-lowering properties of whole cowpea seed and its protein isolate in hamsters. J. Food Sci..

[112] Sinha R., Kawatra A. (2003). Effect of processing on phytic acid and polyphenol contents of cowpeas [*Vigna unguiculata* (L) Walp]. Plant Foods Hum. Nutr..

[113] Feulner G. (2017). Global Challenges: Climate Change. Glob. Chall..

[114] Vermeulen S.J., Campbell B.M., Ingram J.S.I. (2012). Climate change and food systems. Annu. Rev. Environ. Resour..

[115] Schmidhuber J., Tubiello F.N. (2007). Global food security under climate change. Proc. Natl. Acad. Sci. USA.

[116] Kumar B., Bhalothia P. (2020). Orphan crops for future food security. J. Biosci..

[117] Tadele Z. (2019). Orphan crops: Their importance and the urgency of improvement. Planta.

[118] Talabi A.O., Vikram P., Thushar S., Rahman H., Ahmadzai H., Nhamo N., Shahid M., Singh R.K. (2022). Orphan Crops: A best fit for dietary enrichment and diversification in highly deteriorated marginal environments. Front. Plant Sci..

[119] Mabhaudhi T., Chimonyo V.G.P., Hlahla S., Massawe F., Mayes S., Nhamo L., Modi T.A. (2019). Prospects of orphan crops in climate change. Planta.

[120] FAO (2018). Future Smart Food-Rediscovering Hidden Treasures of Neglected and Underutilized Species for Zero Hunger in Asia.

[121] Masipa T.S. (2017). The impact of climate change on food security in South Africa: Current realities and challenges ahead. Jàmbá—J. Disaster Risk Stud..

[122] Egbadzor K.F., Danquah E.Y., Ofori K., Yeboah M., Offei S.K. (2014). Diversity in 118 Cowpea [*Vigna unguiculate* (L.) Walp] Accessions Assessed with 16 Morphological Traits. Int. J. Plant Breed. Genet..

[123] Muñoz-Amatriaín M., Mirebrahim H., Xu P., Wanamaker S.I., Luo M., Alhakami H., Alpert M., Atokple I., Batieno B.J., Boukar O. (2017). Genome resources for climate-resilient cowpea, an essential crop for food security. Plant J..

[124] Horn L.N., Shimelis H. (2020). Production constraints and breeding approaches for cowpea improvement for drought prone agro-ecologies in Sub-Saharan Africa. Ann. Agric. Sci..

[125] Singh A., Singh Y.V., Sharma A., Visen A., Singh M.K., Singh S. (2016). Genetic analysis of quantitative traits in cowpea [*Vigna unguiculata* (L.) Walp.] using six parameter genetic model. Legume Res..

[126] Hall A.E. (2012). Phenotyping cowpeas for adaptation to drought. Front. Physiol..

[127] Rodriguez R.E., Debernardi J.M., Palatnik J.F. (2014). Morphogenesis of simple leaves: Regulation of leaf size and shape. Wiley Interdiscip. Rev. Dev. Biol..

[128] Fritz M.A., Rosa S., Sicard A. (2018). Mechanisms underlying the environmentally induced plasticity of leaf morphology. Front. Genet..

[129] Scoffoni C., Rawls M., Mckown A., Cochard H., Sack L. (2011). Decline of leaf hydraulic conductance with dehydration: Relationship to leaf size and venation architecture. Plant Physiol..

[130] Chitwood D.H., Sinha N.R. (2016). Review Evolutionary and Environmental Forces Sculpting Leaf Development. Curr. Biol..

[131] Okonya J., Maass B. (2014). Protein and iron composition of cowpea leaves: An evaluation of six cowpea varieties grown in Eastern Africa. Afr. J. Food Agric. Nutr. Dev..

[132] El-Shaieny A.A.H., Abdel-Ati Y.Y., El-Damarany A.M., Rashwan A.M. (2015). Stability analysis of components characters in cowpea [*Vigna unguiculata* (L.) Walp]. J. Hortic. For..

[133] Sousa M.B.E., Damasceno-Silva K.J., Rocha M.D.M., De Menezes J.Â.N., Lima L.R.L. (2018). Genotype by environment interaction in cowpea lines using GGE biplot method. Rev. Caatinga.

[134] Mekonnen T.W., Mekbib F., Amsalu B., Gedil M., Labuschagne M. (2022). Genotype by environment interaction and grain yield stability of drought tolerant cowpea landraces in Ethiopia. Euphytica.

[135] Matova P.M., Gasura E. (2018). Yield and stability of new cowpea varieties in Zimbabwe. Afr. Crop Sci. J..

[136] Gerrano A.S., van Rensburg W.S.J., Mathew I., Shayanowako A.I.T., Bairu M.W., Venter S.L., Swart W., Mofokeng A., Mellem J., Labuschagne M. (2020). Genotype and genotype × environment interaction effects on the grain yield performance of cowpea genotypes in dryland farming system in South Africa. Euphytica.

[137] Mbuma N.W., Gerrano A.S., Lebaka N., Mofokeng A., Labuschagne M. (2021). The evaluation of a southern African cowpea germplasm collection for seed yield and yield components. Crop Sci..

[138] Gerrano A.S., Thungo Z.G., Shimelis H., Mashilo J., Mathew I. (2022). Genotype-by-environment Interaction for the contents of micro-nutrients and protein in the green pods of cowpea. Agriculture.

[139] Herniter I.A., Muñoz-Amatriaín M., Lo S., Guo Y.N., Close T.J. (2018). Identification of candidate genes controlling black seed coat and pod tip color in cowpea [*Vigna unguiculata* (L.) Walp]. G3 Genes Genomes Genet..

[140] Herniter I.A., Lo R., Muñoz-Amatriaín M., Lo S., Guo Y.N., Huynh B.L., Lucas M., Jia Z., Roberts P.A., Lonardi S. (2019). Seed coat pattern QTL and development in cowpea [*Vigna unguiculata* (L.) Walp]. Front. Plant Sci..

[141] Huynh B.L., Matthews W.C., Ehlers J.D., Lucas M.R., Santos J.R., Ndeve A., Close T.J., Roberts P.A. (2016). A major QTL corresponding to the Rk locus for resistance to root-knot nematodes in cowpea [*Vigna unguiculata* (L.) Walp]. Theor. Appl. Genet..

[142] Lo S., Muñoz-Amatriaín M., Boukar O., Herniter I., Cisse N., Guo Y.N., Roberts P.A., Xu S., Fatokun C., Close T.J. (2018). Identification of QTL controlling domestication-related traits in cowpea [*Vigna unguiculata* (L.) Walp]. Sci. Rep..

[143] Lonardi S., Muñoz-Amatriaín M., Liang Q., Shu S., Wanamaker S.I., Lo S., Tanskanen J., Schulman A.H., Zhu T., Luo M.C. (2019). The genome of cowpea [*Vigna unguiculata* (L.) Walp]. Plant J..

[144] Elakhdar A., EL-Sattar M.A., Amer K., Rady A., Kumamaru T. (2016). Population structure and marker–trait association of salt tolerance in barley (*Hordeum vulgare* L.). Comptes Rendus. Biol..

[145] González A.M., Yuste-Lisbona F.J., Saburido S., Bretones S., De Ron A.M., Lozano R., Santalla M. (2016). Major contribution of flowering time and vegetative growth to plant production in common bean as deduced from a comparative genetic mapping. Front. Plant Sci..

[146] Xu P., Wu X., Wang B., Hu T., Lu Z., Liu Y., Qin D., Wang S., Li G. (2013). QTL mapping and epistatic interaction analysis in asparagus bean for several characterized and novel horticulturally important traits. BMC Genet..

[147] Rodrigues M.A., Santos C.A., Santana J.R. (2012). Mapping of AFLP loci linked to tolerance to cowpea golden mosaic virus. Genet. Mol. Res..

[148] Pottorff M., Roberts P.A., Close T.J., Lonardi S., Wanamaker S., Ehlers J.D. (2014). Identification of candidate genes and molecular markers for heat-induced brown discoloration of seed coats in cowpea [*Vigna unguiculata* (L.) Walp]. BMC Genom..

[149] Lucas M.R., Ehlers J.D., Roberts P.A., Close T.J. (2012). Markers for the quantitative inheritance of resistance to foliar thrips in cowpea. Crop Sci..

[150] Muchero W., Ehlers J.D., Close T.J., Roberts P.A. (2011). Genic SNP markers and legume synteny reveal candidate genes underlying QTL for Macrophomina phaseolina resistance and maturity in cowpea [*Vigna unguiculata* (L) Walp.]. BMC Genom..

[151] Ouédraogo J.T., Maheshwari V., Berner D.K., St-Pierre C.A., Belzile F., Timko M.P. (2001). Identification of AFLP markers linked to the resistance of cowpea (*Vigna unguiculata* L.) to parasitism by Striga gesnerioides. Theor. Appl. Genet..

[152] Andargie M., Pasquet R.S., Muluvi G.M., Timko M.P. (2013). Quantitative trait loci analysis of flowering time related traits identified in recombinant inbred lines of cowpea (*Vigna unguiculata*). Genome.

[153] Kongjaimun A., Kaga A., Tomooka N., Somta P., Shimizu T., Shu Y., Isemura T., Vaughan D.A., Srinives P. (2012). An SSR-based linkage map of yardlong bean (*Vigna unguiculata* (L.) Walp. subsp. unguiculata Sesquipedalis Group) and QTL analysis of pod length. Genome.

[154] Kongjaimun A., Somta P., Tomooka N., Kaga A., Vaughan D.A., Srinives P. (2013). QTL mapping of pod tenderness and total soluble solid in yardlong bean [*Vigna unguiculata* (L.) Walp. subsp. unguiculata cv.-gr. sesquipedalis]. Euphytica.

[155] Muchero W., Ehlers J.D., Roberts P.A. (2010). QTL analysis for resistance to foliar damage caused by *Thrips tabaci* and *Frankliniella schultzei* (Thysanoptera: Thripidae) feeding in cowpea [*Vigna unguiculata* (L.) Walp.]. Mol. Breed..

[156] Andargie M., Pasquet R.S., Gowda B.S., Muluvi G.M., Timko M.P. (2011). Construction of a SSR-based genetic map and identification of QTL for domestication traits using recombinant inbred lines from a cross between wild and cultivated cowpea [*V. unguiculata* (L.) Walp.]. Mol. Breed..

[157] Guo J., Huang Z., Sun J., Cui X., Liu Y. (2021). Research progress and future development trends in medicinal plant transcriptomics. Front. Plant Sci..

[158] Chen X., Laudeman T.W., Rushton P.J., Spraggins T.A., Timko M.P. (2007). CGKB: An annotation knowledge base for cowpea (*Vigna unguiculata* L.) methylation filtered genomic gene space sequences. BMC Bioinform..

[159] Xia Q., Pan L., Zhang R., Ni X., Wang Y., Dong X., Gao Y., Zhang Z., Kui L., Li Y. (2019). The genome assembly of asparagus bean (*Vigna unguiculata* ssp. *sesquipedialis*). Sci. Data.

[160] Spriggs A., Henderson S.T., Hand M.L., Johnson S.D., Taylor J.M., Koltunow A. (2018). Assembled genomic and tissue-specific transcriptomic data resources for two genetically distinct lines of cowpea [*Vigna unguiculata* (L.) Walp]. Gates Open Res..

[161] Huang K., Mellor K.E., Paul S.N., Lawson M.J., Mackey A.J., Timko M.P. (2012). Global changes in gene expression during compatible and incompatible interactions of cowpea (*Vigna unguiculata* L.) with the root parasitic angiosperm Striga gesnerioides. BMC Genom..

[162] Singh K., Gupta K., Tyagi V., Rajkumar S. (2020). Plant genetic resources in India: Management and utilization. Vavilov J. Genet. Breed..

[163] Mahalakshmi V., Ng Q., Lawson M., Ortiz R. (2007). Cowpea [*Vigna unguiculata* (L.) Walp.] core collection defined by geographical, agronomical and botanical descriptors. Plant Genet. Resour. Charact. Util..

[164] Fatokun C., Girma G., Abberton M., Gedil M., Unachukwu N., Oyatomi O., Yusuf M., Rabbi I., Boukar O. (2018). Genetic diversity and population structure of a mini-core subset from the world cowpea [*Vigna unguiculata* (L.) Walp] germplasm collection. Sci. Rep..

[165] Xiong H., Shi A., Mou B., Qin J., Motes D., Lu W., Ma J., Weng Y., Yang W., Wu D. (2016). Genetic diversity and population structure of cowpea [*Vigna unguiculata* (L.) Walp]. PLoS ONE.

[166] Huynh B.L., Close T.J., Roberts P.A., Hu Z., Wanamaker S., Lucas M.R., Chiulele R., Cissé N., David A., Hearne S. (2013). Gene pools and the genetic architecture of domesticated cowpea. Plant Genome.

[167] Shegro A., Lubinga M.H., Wolday M. (2022). Genetic resources management, seed production constraints and trade performance of orphan crops in Southern Africa: A case of Cowpea. S. Afr. J. Bot..

[168] Tadele Z. (2018). African orphan crops under abiotic stresses. Scientifica.

[169] Kamenya S.N., Mikwa E.O., Song B., Odeny D.A. (2021). Genetics and breeding for climate change in Orphan crops. Theor. Appl. Genet..

[170] Bhat J.A., Ali S., Salgotra R.K., Mir Z.A., Dutta S., Jadon V., Tyagi A., Mushtaq M., Jain N., Singh P.K. (2016). Genomic selection in the era of next generation sequencing for complex traits in plant breeding. Front. Genet..

[171] Marulanda J.J., Mi X., Utz H.F., Melchinger A.E., Würschum T., Longin C.F.H. (2021). Optimum breeding strategies using genomic and phenotypic selection for the simultaneous improvement of two traits. Theor. Appl. Genet..

[172] Varshney R.K., Thudi M., Pandey M.K., Tardieu F., Ojiewo C., Vadez V., Whitbread A.M., Siddique K.H.M., Nguyen H.T., Carberry P.S. (2018). Accelerating genetic gains in legumes for the development of prosperous smallholder agriculture: Integrating genomics, phenotyping, systems modelling and agronomy. J. Exp. Bot..

[173] Pandey M.K., Roorkiwal M., Singh V.K., Ramalingam A., Kudapa H., Thudi M., Chitikineni A., Rathore A., Varshney R.K. (2016). Emerging genomic tools for legume breeding: Current status and future prospects. Front. Plant Sci..

[174] Ye C.Y., Fan L. (2021). Orphan crops and their wild relatives in the genomic era. Mol. Plants.

[175] Leridon H. (2020). World population outlook: Explosion or implosion?. Popul. Soc..

[176] Tilman D., Balzer C., Hill J., Befort B.L. (2011). Global food demand and the sustainable intensification of agriculture. Proc. Natl. Acad. Sci. USA.

[177] Razzaq A., Kaur P., Akhter N., Wani S.H., Saleem F. (2021). Next-generation breeding strategies for climate-ready crops. Front. Plant Sci..

[178] Hussain B. (2015). Modernization in plant breeding approaches for improving biotic stress resistance in crop plants. Turk. J. Agric. For..

[179] Chiurugwi T., Kemp S., Powell W., Hickey L.T. (2019). Speed breeding orphan crops. Theor. Appl. Genet..

[180] Fiyaz R.A., Ajay B.C., Ramya K.T., Kumar A., Sundaram R.M., Rao L.V.S., Singh G.S., Hussain W.S. (2022). Speed Breeding: Methods and Applications in Accelerated Plant Breeding.

[181] Wanga M.A., Shimelis H., Mashilo J., Laing M.D. (2021). Opportunities and challenges of speed breeding: A review. Plant Breed..

[182] Sarsu F. (2021). Contribution of induced mutation in crops to global food security. ACI Av. Cienc. Ing..

[183] Sangle S.M., Lad J.S. (2020). Review on mutation breeding for improvement of food legumes-past and recent. Int. J. Res. Anal. Rev..

[184] Chaudhary J., Deshmukh R., Sonah H. (2019). Mutagenesis approaches and their role in crop improvement. Plants.

[185] Krishnan A., Guiderdoni E., An G., Hsing Y.C., Han C., Lee M.C., Yu S.M., Upadhyaya N., Ramachandran S., Zhang Q. (2009). Mutant resources in rice for functional genomics of the grasses. Plant Physiol..

[186] Horn L.N., Ghebrehiwot H.M., Shimelis H.A. (2016). Selection of novel cowpea genotypes derived through gamma irradiation. Front. Plant Sci..

[187] Zhang L., Li X., Ma B., Gao Q., Du H., Han Y., Li Y., Cao Y., Qi M., Zhu Y. (2017). The tartary buckwheat genome provides insights into rutin biosynthesis and abiotic stress tolerance. Mol. Plant.

[188] Venezia M., Krainer K.M. (2021). Current advancements and limitations of gene editing in orphan crops. Front. Plant Sci..

[189] Østerberg J.T., Xiang W., Olsen L.I., Edenbrandt A.K., Vedel S.E., Christiansen A., Landes X., Andersen M.M., Pagh P., Sandøe P. (2017). Accelerating the domestication of new crops: Feasibility and approaches. Trends Plant Sci..

[190] Kang Y.J., Lee T., Lee J., Shim S., Jeong H., Satyawan D., Kim M.Y., Lee S.H. (2016). Translational genomics for plant breeding with the genome sequence explosion. Plant Biotechnol. J..

[191] Michael T.P., Van Buren R. (2015). Progress, challenges and the future of crop genomes. Curr. Opin. Plant Biol..

[192] Bao A., Zhang C., Huang Y., Chen H., Zhou X., Cao D. (2020). Genome editing technology and application in soybean improvement. Oil Crop Sci..

[193] Pacher M., Puchta H. (2017). From classical mutagenesis to nuclease-based breeding—directing natural DNA repair for a natural end-product. Plant J..

[194] Gbash S., Ade O., Adebiyi J.A., Targuma S., Tebele S., Areo O.M., Olopade B., Odukoya J.O., Njobeh P. (2021). Food safety, food security and genetically modified organisms in Africa: A current perspective. Biotechnol. Genet. Eng. Rev..

[195] Huesing J.E., Romeis J., Ellstrand N.C., Raybould A., Hellmich R.L., Wolt J.D., Dabiré-Binso L.C., Fatokun C.A., Hokanson K.E., Ishiyaku M.F. (2011). Regulatory considerations surrounding the report of the deliberations of an expert. GM Crops.

